# Predictors of colorectal cancer screening intention based on the integrated theory of planned behavior among the average-risk individuals

**DOI:** 10.1186/s12889-022-14191-9

**Published:** 2022-09-22

**Authors:** Mina Maheri, Baratali Rezapour, Alireza Didarloo

**Affiliations:** 1 Social Determinants of Health Research Center, Clinical Research Institute, Urmia University of Medical Sciences, Urmia, 5756115198 Iran; 2 Department of Public Health, School of Public Health, Urmia University of Medical Sciences, Urmia, Iran

**Keywords:** Screening, Colorectal Cancer, Theory of planned behavior, Health action process approach, Average-risk individuals

## Abstract

**Background:**

This study aimed to determine the predictors of colorectal cancer screening intention based on the integrated theory of planned behavior among average -risk individuals in Urmia. Identifying these predictors will help design and implement various interventions, including educational interventions, according to the needs of this group, thereby taking a step towards improving the colorectal cancer screening index.

**Methods:**

The present cross-sectional study was performed on 410 individuals at average risk of colorectal cancer referring to the comprehensive health services centers of Urmia in Iran. The data collection tool was a researcher-made questionnaire consisting of two parts. The first part captured the demographic information and medical history of the participants. The second part involved questions designed based on constructs of motivational phase of health action process approach, and theory of planned behavior, as well as behavioral intent to perform colorectal cancer screening. Data analysis was performed using SPSS software.

**Results:**

Outcome expectancies, risk perception, action self-efficacy, and normative beliefs, respectively had the largest impact and were significant and positive predictors of colorectal cancer screening intention. The study’s conceptual framework explained about 36% of the variance of behavioral intention among the average-risk individuals in Urmia.

**Conclusions:**

Constructs of motivational phase of health action process approach, and theory of planned behavior are valuable and appropriate to identify the factors affecting the intention to undergo colorectal cancer screening as well as to design and implement educational interventions in this field. The four constructs of outcome expectancies, risk perception, action self-efficacy, and normative beliefs are suggested to be integrated into all educational interventions designed and implemented to improve the colorectal cancer screening index.

## Introduction

Colorectal Cancer (CRC) is currently the third leading cause of cancer death globally, accounting for about 9% of cancer deaths [1]. According to 2018 data, CRC is the third most prevalent cancer worldwide, claiming 11% of cancer diagnoses. The number of new cases of this disease in 2018 was 1.8 million [1]. In Iran, CRC is the third most common cancer among men and the fourth most common cancer among women. The prevalence, incidence, and death rate of CRC in Iran are increasing due to lifestyle changes (including unhealthy diet and decreased physical activity) as well as low participation in screening programs [2].

Given the shocking prevalence and death rates of CRC, secondary prevention of this disease is as important as primary prevention (such as having a healthy lifestyle) [1, 3]. With secondary prevention, which indeed refers to early detection using screening tests, essential measures can be taken for rapid treatment and prevention of cancer progression [1, 3]. Regarding CRC screening, it is necessary to mention that CRC screening not only leads to early detection of existing CRC, but can also prevent CRC by finding pre-cancerous polyps that can be removed [3].

In population-based CRC screening programs, the immunochemical fecal occult blood test (iFOBT), also called fecal immunochemical test (FIT), is superior to other CRC screening tests due to its ease and low cost [4–6]. In the Iranian healthcare system, the CRC screening program for average-risk individuals^1^ follows a global pattern [7]. Accordingly, it is recommended for average risk individuals have an FIT once a year, and if the test is positive, these individuals are referred for additional tests, including colonoscopy [8, 9]. Although screening tests for CRC are available in Iran, the majority of people are not informed of their cancer risk or the available screening tests, and never receive a physician recommendation for screening [8]. Also, in the study conducted by Javadzade et al., the lack of information, fear of cancer diagnosis, and lack of recommendation by doctors were identified as barriers related to colorectal cancer screening [10]. Despite the effectiveness of screening programs in diagnosing early and treatable cancers, these factors cause many high-risk individuals not to participate in CRC screening programs [11, 12].

Thus, identifying the important factors affecting the CRC screening intention among the average risk individuals will provide health system policymakers and practitioners with the opportunity and ability to design various interventions, including educational interventions, according to the needs of this group; in this way, a step will be taken to improve the CRC screening index. In the meantime, theories and models of health education can help researchers determine the factors affecting the intention and adoption of health behaviors [13]. Similarly, applying these models and theories makes it possible to identify barriers to participation in screening programs and improve the CRC screening index by controlling or removing these barriers [14]. According to the mentioned points, the present study was conducted to determine the predictors of CRC screening intention based on the constructs of motivational phase of health action process approach and theory of planned behavior among the average-risk individuals in Urmia.

### Conceptual framework of study

Since the aim of the present study is to determine the predictors of the intention to perform CRC screening, models and theories that explain and predict the behavioral intention such as health action process approach (HAPA) and the theory of planned behavior (TPB) will be useful and practical. Based on the literature review, no previous study seems to have been conducted in the field of CRC screening with the combination of TPB and HAPA constructs; however, other studies have indicated the effectiveness of the combination of TPB and HAPA constructs in explaining and predicting the intention to perform health behaviors [15, 16]. For example, in the study conducted by Zhang et al. [15], the effectiveness of the combination of TPB and HAPA has been confirmed in predicting hand washing and sleep hygiene behaviors. They recommended the combined use of these two models to predict the intention to perform health behaviors as well as to design educational interventions with the aim of improving the intention to perform health behaviors.

The HAPA is one of the theories that has helped better understand the factors affecting the change of intention and behavior [17]. In this model, changing health behavior consists of two phases (motivational and volitional). In the motivational phase, three factors of risk perception, outcome expectancies, and action self-efficacy influence the behavioral intention formation and prepare the individual to accept certain behaviors as well as related decisions. However, one of the limitations of this approach is that ignoring social factors affects the formation of behavioral intention [17]. Thus, combining this approach with the TPB will compensate for this limitation, since the TPB with its construct of subjective norms in addition to individual factors, also considers social factors affecting the behavioral intention to some extent [13, 17]. TPB is one of the most common theories in the area of health behavior change. According to this theory, the most critical factor in determining a person’s behavior is behavioral intention, where determinants of behavioral intention are three factors: attitude, subjective norms, and perceived behavioral control [13]. According to the given explanations, the motivational phase of the HAPA and TPB were chosen as the conceptual framework of the present study.

### Study variables

Independent variables: constructs of motivational phase of HAPA including risk perception, outcome expectancies, and action self-efficacy as well as indirect constructs of TPB including behavioral beliefs and outcome evaluation (determinants of the attitude construct), normative beliefs and motivation to comply (determinants of the subjective norms construct), and control beliefs and perceived power (determinants of the perceived behavioral control construct).

#### Dependent variable: behavioral intention

##### Operational definition of the study variables

-Risk perception refers to participants’ subjective assessments of the risk of developing CRC and severity of CRC as well as its potential consequences. As the risk perception toward CRC increases, so do the intention and likelihood of undergoing the CRC screening.
-Outcome expectancies refer to participants’ subjective assessments of the possible positive plus negative consequences of CRC screening. As the perception of positive consequences of CRC screening increases, so do the intention and likelihood of undergoing the CRC screening.
-Action self-efficacy refers to the participants’ beliefs in their ability to initiate CRC screening. As the action self-efficacy toward CRC screening increases, so do the intention and likelihood of undergoing the CRC screening.
-Attitude refers to the participants’ overall feelings of like or dislike toward CRC screening. As the feelings of like toward CRC screening increases, the intention and likelihood of doing the CRC screening also grow. Attitude is determined by two indirect constructs: behavioral beliefs and outcome evaluation.
-Behavioral beliefs refer to participants’ subjective assessments of the possible positive and negative consequences of CRC screening (equivalent to outcome expectancies).
-Outcome evaluation refers to the value participants place on each of the possible positive and negative consequences of CRC screening. As the value of possible positive consequences of CRC screening increases, the intention and likelihood of undergoing the CRC screening also rise.
-Subjective norms refer to participants’ beliefs that significant others in their life, think they should or should not perform the behavior. As the participants’ beliefs that significant others in their life, think they should do the CRC screening increases, the intention and likelihood of undergoing the CRC screening also increase. Subjective norms are determined by two indirect constructs: normative beliefs and motivation to comply.
-Normative beliefs refer to how participants’ thinks about the significant others in their life, whether they would like them to do CRC screening or not. As the participants’ thoughts about the significant others in their life increase in that they would like them to undergo CRC screening, so do the intention and likelihood of doing the CRC screening.
-Motivation to comply refers to the degree to which participants want to act in accordance with the wishes of significant others in their life. As the desire to act in accordance with the wishes of significant others in their life increases (and if one of their wishes is CRC screening), so do the intention and likelihood of undergoing the CRC screening.
-Perceived behavioral control refers to participants’ perceptions of their ability to do CRC screening. As the perceptions of ability to do CRC screening increases, the intention and likelihood of undergling the CRC screening also rise. Perceived behavioral control is determined by two indirect constructs: control beliefs and perceived power.
-Control beliefs refer to participants’ beliefs about the internal or external factors that may inhibit or facilitate the CRC screening. As the participants’ beliefs about the internal or external factors that may facilitate the CRC screening increases, so do intention and likelihood of doing the CRC screening. As the participants’ beliefs about the internal or external factors that may inhibit the CRC screening increases, the intention and likelihood of undergoing the CRC screening diminish.
-Perceived power refers to participants’ beliefs of how easy or difficult it is for them to CRC screening despite the facilitators and barriers. As the participants’ beliefs that doing the CRC screening is easy increases, so do the intention and likelihood of undergoing the CRC screening. As the participants’ beliefs that doing the CRC screening is difficult increases, the intention and likelihood of doing the CRC screening decreases.
-Behavioral intention refers to participants’ decisions and intentions to do CRC screening. As the intention to do CRC screening increases, so does the rate of undergoing CRC screening.

## Methods

This descriptive-analytical cross-sectional study was conducted on 410 average risk individuals of CRC who were referred to comprehensive health services centers in Urmia, Iran, 2021. The inclusion criteria included individuals aged 50 to 69 years with an average risk of CRC, physical and mental ability to answer questions, and consent to participate in the study. Exclusion criteria were incomplete completion of the questionnaire.

The minimum sample size required was determined 338 individuals according to a previous similar study and considering the standard deviation of 0.75 for the mean score of CRC screening [18], 95% confidence level (z = 1.96), maximum margin of error or precision (d = 0.08), and using the sample size determination formula for estimating a single mean. Then, to enhance the study power, the number of samples was finally considered 410 individuals.

$$n=\frac{Z_{1- {\propto }\!\left/ \ {2}\right.}^2{S}^2}{d^2}=\frac{1.96^2\ {0.75}^2}{0.08^2}=338$$

A multi-stage cluster sampling method was used for the sampling. First, the city of Urmia was divided into four geographical regions of north, south, east, and west. Then, an urban comprehensive health service center was selected from each region using a simple random sampling method and by lot. Next, by referring to the selected centers and coordinating with the head of the centers, the required samples were completed in proportion to the number of individuals referring to each selected center, from among the individuals who met the inclusion criteria and consented to contribute, via convenience sampling method.

In order to determine whether an individual is at average risk for CRC or not, when going to the health centers for sampling, the information of the health records of the samples available in the centers, as well as the information of the health staff of the centers were used. Also, before completing the questionnaires, the subjects themselves were also asked about the inclusion criteria, and finally, once that an individual was found to be at average risk for CRC and met the other inclusion criteria, he/she was enrolled into the study.

The data collection tool was a researcher-made questionnaire consisting of two parts. The first part captured demographic information and the medical history of participants. The second part involved questions designed based on constructs of motivational phase of HAPA (including risk perception, outcome expectancies and action self-efficacy), and TPB (including behavioral beliefs, outcome evaluation, normative beliefs, motivation to comply, control beliefs, and perceived power), as well as behavioral intention to undergo CRC screening.

The initial questions of the researcher-made questionnaire were designed based on a literature review and opinions of experts in fields related to research and scale development, after which its validity and reliability were measured and approved. In order to determine the validity, two methods of face validity (qualitative and quantitative type) and content validity (quantitative type) were used.

In the qualitative face validity, 20 individuals from the target group were interviewed face to face. They were asked about the suitability and proper relevance of the questions with each other and with the related construct, difficulty in understanding the words, phrases, and statements, as well as possibility of ambiguity and misinterpretations regarding the meanings of words, phrases, and statements. If there was a problem, their opinions would be taken and included in the questionnaire [19].

In the quantitative face validity, the impact score was calculated for each question. For this purpose, a panel of experts was employed, where the questionnaire was given to 10 experts in fields related to research and scale development (including 6 Health education specialists, 2 Epidemiologist, 1 Gastroenterologist, and 1 General surgeon); they were asked to assign each question a score of 1 to 5 in terms of their importance. A score of 1 indicates the lowest, while a score of 5 represents the highest importance. Questions with an impact score greater than 1.5 were deemed suitable for further analysis and remained in the questionnaire; otherwise, they were excluded [19].

In the quantitative content validity, the prepared pilot questionnaire was provided to the panel of experts mentioned above, where the content validity ratio (using the criterion of essentiality) and content validity index (using the relevance, clarity, and simplicity criteria) were calculated. Questions with a content validity ratio of greater than 0.62 and a content validity index of larger than 0.79 were accepted [19].

Cronbach’s alpha coefficient was used to assess the reliability of the researcher-made questionnaire. For this purpose, the prepared pilot questionnaire was given to 30 people in the target group, and after completing the questionnaires, Cronbach’s alpha coefficient was calculated. For all constructs, Cronbach’s alpha coefficient was above 0.7, so the reliability of the tools used in this study was optimal [19].

CVR, CVI, and Cronbach’s alpha were 0.916, 0.959, and 0.942, respectively, for risk perception constructs. For other constructs, the following were obtained: outcome expectancies (0.895, 0.934 and 0.832), outcome evaluation (0.895, 0.934 and 0.824), action self-efficacy (0.942, 0.970 and 0.946), normative beliefs (0.875, 0.913 and 0.925), motivation to comply (1, 1 and 0.820), control beliefs (0.847, 0.924 and 0.888), perceived power (0.847, 0.924 and 0.836), and behavioral intention (0.916, 0.927 and 0.912).

The initial questionnaire involved 111 construct questions, which decreased to 100 questions after dealing with validity and reliability. The final questionnaire included 12 questions associated with the construct of risk perception, 12 questions with outcome expectancies, 12 with outcome evaluation, 13 with action self-efficacy, 8 with normative beliefs, 4 with motivation to comply, 18 with control beliefs, 18 with perceived power, and three questions related to behavioral intention. Possible answers to constructs of motivational phase of HAPA and TPB were scored in 5-point Likert including strongly disagrees (1), somewhat disagrees (2), have no opinion (3), somewhat agree (4) and strongly agree (5). In general, obtaining a higher score in each construct would indicate a good condition of the subject in terms of the understudy construct. The questionnaires were completed by trained interviewers and through self-reporting technique.

Ethical considerations of the present study included receiving the ethics’ code from the research ethics committee of the Vice Chancellor for Research & Technology of Urmia University of Medical Sciences (IR.UMSU.REC.1398.201), receiving a written letter of introduction from relevant authorities to present to research environments, the presence of researchers in selected centers and stating the objectives of the study, obtaining informed consent from the volunteers to participate in the study, presenting sufficient explanation to them about the purpose of the study and the method of work, as well as assuring them that their participation in the study was entirely voluntary. If they did not wish to either participate or continue, they could withdraw from the study, and their information would be kept confidential by the researcher, and the study results would be reported only in general. The questionnaire had no first or last name.

Finally, the data obtained were analyzed in SPSS software version 23 using descriptive statistics (mean, standard deviation, min, max, percentage, and frequency) and analytical statistics including Kolmogorov-Smirnov (to check the normality of the data), Independent t-test (to compare the mean score of CRC screening intention among the two independent groups of the participants), One-way ANOVA (to compare the mean score of CRC screening intention among the three or more independent groups of the participants), Pearson correlation coefficient (to determine the degree of linear correlation between CRC screening intention and the independent variable), and Multiple linear regression with Enter method (to determine the predictive power of the constructs of motivational phase of HAPA and TPB on the CRC screening intention). The results were considered statistically significant at *p* < 0.05.

## Results

Table 1 summarizes the status of demographic characteristics and medical history of research units. According to the findings, the mean age of the subjects was 58.60 ± 5.52 years. The majority of research units were female (54.1%), married (81.2%), with an elementary education level (23.7), housewife (40.5%) and government (32.7%) employee status, and with a medium economic status (56.8%). Only 10.7% reported having a history of FIT.

**Table 1:** Demographic and clinical characteristics of participants (*N* = 410)

Variables	Sub-variables	n(%)	Variables	Sub-variables	n(%)
Gender	Male	188 (45.9)	History of specific physical illness	Yes	188 (45.9)
	Female	222 (54.1)		No	222 (54.1)
Marital status	Single	12 (2.9)	Using special drugs experience	Yes	220 (53.7)
	Married	333 (81.2)		No	190 (46.3)
	Widow	65 (15.9)	Covered by medical insurance	Yes	368 (89.8)
Educational status^a^	Illiterate	88 (21.5)		No	42 (10.2)
	Elementary	97 (23.7)	History examinations and tests for CRC such as Colonoscopy, Sigmoidoscopy, etc.	Yes	53 (12.9)
	Middle school	62 (15.1)		No	357 (87.1)
	High school & Diploma	78 (19.0)	History of iFOBT (FIT)	Yes	44 (10.7)
	University	85 (20.7)		No	366 (89.3)
Employment status	Unemployed	14 (3.4)	General health status^c^	Excellent	14 (3.4)
	Housewife	166 (40.5)		very good	30 (7.3)
	Government employee	134 (32.7)		Good	166 (40.5)
	Self-employed	96 (23.4)		Fair	167 (40.7)
Family income economic status^b^	Low	52 (12.7)		Poor	33 (8.1)
	Medium	233 (56.8)	Housing status	Landlord	339 (82.7)
	Good	106 (25.9)		Tenant	71 (17.3)
	Excellent	19 (4.6)			
**Variables**	**Mean**	**±SD**	**Variables**	**Mean**	**±SD**
Age(year)	58.70	5.52	Number of family members	3.28	1.70

*Abbreviations*: *n* number, *SD* Standard deviation, *iFOBT* Immunochemical fecal occult blood test, *FIT* Fecal immunochemical test

^a^Educational status was measured based on the number of years of education and an illiterate person means someone who has no years of education

^b^Economic status was measured based on the individual’s perception of their economic status and income

^c^General health status was measured based on the individual’s perception of their general health status

Table 2 presents the mean scores of constructs of motivational phase of HAPA (including risk perception, outcome expectancies, and action self-efficacy), constructs of TPB (including behavioral beliefs, outcome evaluation, normative beliefs, motivation to comply, control beliefs, and perceived power), and behavioral intention to undergo CRC screening among the participants. The lowest mean score was related to the construct of control beliefs and perceived power (obtaining about 59 out of 100 points), while the highest mean was related to the construct of risk perception (obtaining about 72 out of 100 points).

**Table 2:** Mean scores of motivational phase constructs of the HAPA and TPB constructs (*N* = 410)

Variables	Constructs	Mean ± SD	Scale range^a^	Min-Max^b^	Mean score (Out of 100)
Motivational phase constructs of the HAPA	Risk perception	43.57 ± 9.42	12-60	12-60	72.62 ± 15.70
	Outcome expectancies (equivalent to behavioral beliefs)^c^	36.40 ± 7.18	12-60	13-56	60.67 ± 11.97
	Action self-efficacy	40.66 ± 10.84	13-65	13-65	62.55 ± 16.68
TPB constructs	behavioral beliefs(equivalent to Outcome expectancies)	36.40 ± 7.18	12-60	13-56	60.67 ± 11.97
	Outcome evaluation	40.76 ± 6.96	12-60	18-58	67.93 ± 11.60
	Normative beliefs	26.54 ± 6.22	8-40	9-40	66.37 ± 15.55
	Motivation to comply	13.90 ± 3.64	4-20	4-20	69.54 ± 18.20
	Control beliefs	53.35 ± 11.50	18-90	27-80	59.27 ± 12.78
	Perceived power	53.50 ± 12.89	18-90	18-84	59.44 ± 14.33
	Behavioral intention	9.18 ± 2.99	3-15	3-15	61.20 ± 19.98

*Abbreviations*: *SD* Standard deviation

^a^The lowest and highest values that can be obtained from the original scale

^b^The lowest and highest values obtained in this study

^c^Outcome expectancies are equivalent to behavioral beliefs, and both refer to person’s belief that performing a given behavior will lead to certain outcomes

The mean scores of the behavioral intention to undergo CRC screening according to the demographic characteristics, and the subjects’ medical history are reported in Table 3. The results of the ANOVA test indicated that there is a statistically significant relationship between education level and behavioral intention. Then, using the Bonferroni test, the differences between different educational groups were examined in pairs. According to the findings, the mean score of behavioral intention was lower among illiterate people than those with higher education, including elementary (*p* < 0.001) and university (*p* = 0.029). The results of the ANOVA test also revealed that there is a statistically significant relationship between family economic status and behavioral intention. Based on the Bonferroni test results, the mean score of behavioral intention was higher among people with good incomes than people with low (*p* = 0.013), middle (*p* < 0.001), and even excellent incomes (*p* = 0.002).

**Table 3:** Mean score of behavioral intention according to the characteristics of participants (*N* = 410)

Variables	Sub-variables	Mean ± SD	Variables	Sub-variables	Mean ± SD
Gender	Male	9.11 ± 3.24	History of specific physical illness	Yes	9.59 ± 2.80
	Female	9.23 ± 2.77		No	8.83 ± 3.11
	p^a^	0.694		p^a^	0.011
Marital status	Single	10.83 ± 4.40	Using special drugs experience	Yes	9.59 ± 2.80
	Married	9.11 ± 2.97		No	8.70 ± 3.14
	Widow	9.21 ± 2.75		p^a^	0.003
	p^b^	0.148	Covered by medical insurance	Yes	9.29 ± 2.98
Educational status	Illiterate ^ab^	8.44 ± 2.95		No	8.19 ± 2.97
	Elementary ^ac^	10.04 ± 2.36		p^a^	0.024
	Middle school ^c^	8.56 ± 3.77	History examinations and tests for CRC such as Colonoscopy, Sigmoidoscopy, etc.	Yes	10.20 ± 3.29
	High school & Diploma	9.16 ± 3.11		No	9.02 ± 2.29
	University ^b^	9.42 ± 2.69		p^a^	0.007
	p^b^	0.002	History of iFOBT (FIT)	Yes	10.72 ± 2.60
Employment status	Unemployed	8.71 ± 3.02		No	8.99 ± 2.99
	Housewife	9.24 ± 2.76		p^a^	< 0.001
	Government employee	9.28 ± 3.18	General health status	Excellent	8.28 ± 3.07
	Self-employed	9.00 ± 3.13		very good	8.00 ± 3.62
	p^b^	0.823		Good	9.56 ± 2.98
Family income economic status	Low ^d^	8.82 ± 3.02		Fair	9.14 ± 2.67
	Medium ^e^	8.97 ± 3.06		Poor	8.87 ± 3.68
	Good ^def^	10.07 ± 2.71		p^b^	0.060
	Excellent ^f^	7.73 ± 2.46	Housing status	Landlord	9.20 ± 2.97
	p^b^	< 0.001		Tenant	8.94 ± 3.11
				p^a^	0.517

Same alphabet letters demonstrate a statistically significant difference between the two groups based on the Bonferroni correction method

^a^Independent T-test; ^b^One-way ANOVA

The Independent T-test results showed that the mean score of behavioral intention was significantly higher among people with a history of physical illness compared to people without it (*p* = 0.011), people with a history of taking a particular drug compared to those without it (*p* = 0.003), people covered with health insurance compared to those who were not (*p* = 0.024), people who had a history of undergoing examinations and tests related to the colon by a specialist compared to those with no such experience ((*p* = 0.007), as well as people who had a history of FIT compared to those who did not have this history (*p* < 0.001) (Table 3).

Since the correlation coefficient is the basis of causal relationship analysis, before performing the multiple linear regression test, the relationship between the studied constructs with behavioral intention was investigated using the Pearson correlation test [20]. The results indicated a positive and significant correlation between the mean scores of the studied constructs (except for perceived power) and the mean score of behavioral intention (*p* < 0.001). Indeed, upon increase in the scores of risk perception outcome expectancies, action self-efficacy, outcome evaluation, normative beliefs, motivation to comply, and control beliefs among the average-risk individuals for CRC in Urmia, behavioral intention score also increased for CRC screening (Table 4).

**Table 4:** Correlation coefficient between motivational phase constructs of the HAPA, TPB constructs and age with behavioral intention (*N* = 410)

Variable	r	p^a^
Risk perception	0.476	0.001>
Outcome expectancies (equivalent to Behavioral beliefs)	0.440	0.001>
Action self-efficacy	0.465	0.001>
Behavioral beliefs (equivalent to Outcome expectancies)	0.440	0.001>
Outcome evaluation	0.397	0.001>
Normative beliefs	0.405	0.001>
Motivation to comply	0.209	0.001>
Control beliefs	0.391	0.001>
Perceived power	0.038	0.449
Age	0.015	0.768

^a^Pearson correlation

Tables 5 and 6 report the regression coefficients of behavioral intention predictors for CRC screening among the average-risk individuals of CRC in Urmia based on the constructs of motivational phase of HAPA and TPB.

**Table 5:** Predictors of behavioral intention among the participants according to motivational phase constructs of the HAPA and TPB constructs (*N* = 410)

Independent variables	B	SE	*β*	t (*p*)	R^2^	Adjusted R^2^	F (*p*)
Risk perception	0.081	0.018	0.255	4.505(< 0.001)	0.358	0.345	27.969 (< 0.001)
Outcome expectancies (equivalent to Behavioral beliefs)	0.100	0.022	0.240	4.482(< 0.001)			
Action self-efficacy	0.049	0.016	0.179	3.040 (0.003)			
Behavioral beliefs (equivalent to Outcome expectancies)	0.100	0.022	0.240	4.482(< 0.001)			
Outcome evaluation	−0.047	0.028	−0.109	−1.685 (0.093)			
Normative beliefs	0.075	0.025	0.155	3.032 (0.003)			
Motivation to comply	−0.017	0.038	− 0.020	−0.439 (0.661)			
Control beliefs	0.021	0.014	0.081	1.530 (0.127)			
Perceived power	0.006	0.011	0.028	0.613 (0.540)

**Table 6:** Predictors of behavioral intention^a^ among the participants according to motivational phase constructs of the HAPA and TPB constructs (*N* = 410)

Independent variables	B	SE	*β*	t (*p*)	R^2^	Adjusted R^2^	F (*p*)
Risk perception	0.073	0.018	0.230	3.973(< 0.001)	0.386	0.364	17.725 (< 0.001)
Outcome expectancies (equivalent to Behavioral beliefs)	0.097	0.023	0.233	4.291(< 0.001)			
Action self-efficacy	0.056	0.016	0.202	3.401(< 0.001)			
Behavioral beliefs (equivalent to Outcome expectancies)	0.097	0.023	0.233	4.291(< 0.001)			
Outcome evaluation	−0.038	0.028	−0.089	−1.353 (0.177)			
Normative beliefs	0.088	0.025	0.182	3.529(< 0.001)			
Motivation to comply	−0.006	0.038	−0.007	−0.155 (0.877)			
Control beliefs	0.024	0.014	0.094	1.771 (0.077)			
Perceived power	0.009	0.011	0.037	0.820 (0.413			

^a^ Adjusted variables: age, gender, marital status, educational status, and family income economic status

Based on the findings of the adjusted regression coefficient table, outcome expectancies (β = 0.233, *p* < 0.001), risk perception (β = 0.230, *p* < 0.001), action self-efficacy (β = 0.202, *p* < 0.001), and normative beliefs (β = 0.182, *p* < 0.001), respectively had the largest impact, and were significant as well as positive predictors of colorectal cancer screening intention. This means that for one unit increase in outcome expectancies score, the CRC screening intention score rose by about 0.233 unit. Other predictors can also be interpreted in this way (Table 6).

Constructs of motivational phase of HAPA and TPB explained in total about 36% of the variance of CRC screening intention among the average-risk individuals in Urmia (Table 5).

## Discussion

Based on the present study’s findings, the participation rate of the subjects in the CRC screening program was low. Only 10.7% of the participants reported a history of FIT. In a study conducted by Emadi Azam et al., the participation rate of average-risk individuals in CRC screening programs was reported 22.8%, which is higher than the present study’s findings [21]. However, in the Emadi Azam study, the target group consisted of medical professionals, while in the present study, the target group has been the general population, so given the nature of the target groups in these two studies, this finding is not unexpected.

Also, in a study conducted by Besharti et al., among the general population, the rate of this index was reported 7.6%, which is lower than the findings of the present study [22]. Given that in the present study and other studies handled in Iran [21, 22], the participation rate of average-risk individuals in CRC screening programs has been reported as low, this situation is not acceptable, and there is a need to design and implement various interventions to improve this index. Hence, by identifying the important factors affecting the CRC screening intention among average-risk individuals, the present study’s findings will provide the opportunity and ability for health system policymakers and implementers to design as well as implement various interventions, including educational interventions, according to the needs of this group thereby improving the CRC screening index.

Based on the present study’s findings, the construct of outcome expectancies was the strongest predictor of CRC screening among the subjects. In other words, if a person believes that CRC screening has positive results for him/her, he/she will be more willing to undergo it and vice versa. In other studies, the belief in positive results and advantages of CRC screening has been cited as a positive and significant predictor of behavioral intention and CRC screening behavior [21–23]. Thus, it is suggested that strategies related to improving the construct of outcome expectancies be included in all educational interventions designed and implemented to enhance the CRC screening intention. One of the strategies that can effectively improve the construct of outcome expectancies is holding group discussion sessions on the positive consequences of behavior [13]. As such, expressing the positive consequences of CRC screening (such as early detection and treatment of CRC, prevention of cancer progression, reducing the costs associated with cancer treatment) [22, 23] in the form of group discussions can enhance people’s intention to undergo CRC screening.

The construct of risk perception (a combination of perceived susceptibility and perceived severity) was another significant predictor of the intention CRC screening intention among average-risk individuals in Urmia. With increase in a person’s belief in vulnerability and the likelihood of developing CRC, as well as severe complications of this cancer, his/her intention to undergo CRC screening was enhanced, and vice versa. In line with the present study’s findings, in other studies, the individual’s belief in the vulnerability and severity of complications of CRC has been cited as a positive and significant predictor of CRC screening [24, 25].

Contrary to the present study’s findings, in studies conducted by Lin et al. [23] and Zheng et al. [26], vulnerability and susceptibility to CRC as well as the severity of its complications did not predict CRC screening. These different findings can be due to differences in the nature and characteristics of the statistical population under study and the number of samples. Thus, further studies in this field and, of course, with more samples are recommended to obtain more accurate findings.

However, risk perception was an essential predictor of CRC screening among average-risk individuals in Urmia. Hence, emphasizing the possibility of developing CRC in any one and emphasizing the seriousness and severity of its complications in educational programs could expectedly encourage people to undergo CRC screening.

Based on the present study’s findings, action self-efficacy was another predictor of CRC screening. Upon increase in a person’s belief in in his / her ability to undergo colorectal cancer screening, his/her intention to undergo colorectal cancer screening increased and vice versa.

Note that in most studies conducted to determine the predictors of CRC screening based on theories and models in health education, belief in the positive consequences and benefits of CRC screening as well as perceived self-efficacy for screening has been cited as a positive and meaningful predictor [21, 23]. Thus, it is suggested that these two constructs be integrated in all interventions designed and implemented to improve the CRC screening index.

Strategies that can indirectly enhance people’s colorectal cancer screening intention by promoting their perceived self-efficacy include training screening steps in small and simple steps (for example step-by-step training of preparing fecal sample by individuals themselves), introducing individuals whose cancer had been diagnosed and treated early through CRC screening (credible role model), verbal persuasion and reassurance, and relaxation techniques training to reduce the stress of CRC screening [13, 27].

The constructs of normative beliefs constituted another predictor of the colorectal cancer screening intention among participants. In other words, if the people influencing the average-risk individual (including spouse, children, friends, health care staff, etc.) have a positive attitude towards CRC screening and encourages them to do so, person’s intention to undergo colorectal cancer screening will increase. In line with the present study’s findings in other studies, the recommendations of family members, friends, and health care personnel had increased the individual’s intention to undergo colorectal cancer screening [14, 28].

Thus, it is suggested that in addition to average-risk individuals, educational interventions in the field of CRC screening be designed and implemented for people who influence them, including family members, friends, health care staff, etc.

Since based on the literature review, no previous study seems to have been conducted with this title, the present study can be a basis for future studies especially interventional studies. They can be designed and implemented to improve the CRC screening index among average-risk individuals. One of the limitations of the present study was that the data were collected by a self-report method, so there was possibility that participants may have not given true answers to the questions. Also, due to the study’s cross-sectional nature, the relationships found between the variables may be considered causal relationships with due caution.

## Conclusion

The present study’s findings revealed that the four constructs of outcome expectancies, risk perception, action self-efficacy, and normative beliefs were the positive and significant predictors of CRC screening among the average-risk individuals in Urmia. Thus, it is suggested that these four constructs be integrated in all educational interventions designed and implemented to improve the CRC screening index.

Constructs of motivational phase of HAPA and TPB explained in total about 36% of the variance of CRC screening intention among the average-risk individuals in Urmia. Hence, according to the classification of the coefficient of determination (R^2^) in the linear regression test as low (0.02 moderate (0.13), and strong (0.26) [29], it can be concluded that the conceptual framework used in the present study is useful and appropriate to identify the factors affecting the CRC screening intention as well as to design and implement related educational interventions in this field.

Note that any macro-level planning to improve the CRC screening index will be effective if, in addition to individual factors influencing behavioral intention (including outcome expectancies, risk perception, action self-efficacy, normative beliefs), other influential factors such as social, cultural, economic, and enabling factors (such as skills, money, time, equipment and facilities) are also considered.

## Data Availability

The datasets used and/or analysed during the current study are available from the corresponding author on reasonable request.

## Abbreviations

CRC: Colorectal Cancer
iFOBT: Immunochemical Fecal Occult Blood Test
FIT: Fecal Immunochemical Test
HAPA: Health Action Process Approach
TPB: Theory of Planned Behavior
SPSS: Statistical Package for the Social Sciences

## Footnotes

1 - Average-risk individuals are asymptomatic individuals 50 years old or older without colorectal cancer or adenomatous polyps personal experience, without inflammatory bowel disease personal experience, without colorectal cancer family experience in a first-class relative who has been diagnosed before 60 years old or in two first-degree relatives who are diagnosed at any age, and without adenomatous polyp family experience which is diagnosed in a first-degree relative before 60 years old [7, 8].
